# The Unseen Aftermath: Associations Between the COVID-19 Pandemic and Shifts in Mortality Trends in Japan

**DOI:** 10.3390/ijerph22010074

**Published:** 2025-01-08

**Authors:** Hasan Jamil, Shuhei Nomura, Stuart Gilmour

**Affiliations:** 1 Graduate School of Public Health, St. Luke’s International University, Tokyo 104-0045, Japan; sgilmour@slcn.ac.jp; 2 Division of Population Data Science, National Cancer Center Institute for Cancer Control, Tokyo 104-0045, Japan; 3 Keio University Global Research Institute (KGRI), Tokyo 108-8345, Japan; s-nomura@keio.jp; 4 Department of Health Policy and Management, School of Medicine, Keio University, Tokyo 160-8582, Japan

**Keywords:** COVID-19, Japan, non-communicable diseases, mortality trends, pandemic, public health, healthcare systems, gender disparities, epidemiology, health policy

## Abstract

The COVID-19 pandemic disrupted healthcare systems globally, potentially altering mortality trends for non-COVID-19 diseases, particularly in aging populations like Japan’s. Assessing these impacts is essential for responsive healthcare planning. We analyzed Japanese vital registration mortality records from January 2018 to December 2021 for adults aged 25 and older, excluding COVID-19-related deaths. Data were stratified by sex and ICD-10 cause-of-death chapters. Poisson regression models assessed changes in mortality rates and trends, incorporating pandemic-related variables and interactions between time, age group, and the pandemic term. Among the 4,920,942 deaths analyzed, 2,456,750 occurred during the pandemic years. Significant sex-specific changes in mortality trends were observed. Women experienced increases in mortality rates and trends for endocrine, nutritional, and metabolic diseases; skin and subcutaneous tissue diseases; circulatory diseases; and genitourinary diseases, reversing some pre-pandemic declines. Men showed increases in mortality trends for endocrine, nutritional, and metabolic diseases and genitourinary diseases but no significant changes for skin or circulatory diseases. These findings indicate that the pandemic differentially affected mortality trends between sexes, with women experiencing broader increases across multiple disease categories. The COVID-19 pandemic was associated with significant changes in mortality trends for certain non-COVID-19 diseases in Japan, with notable sex differences. Increased mortality among women across multiple disease categories highlights the pandemic’s indirect health impacts and underscores the need for sex-specific healthcare strategies in the post-pandemic era.

## 1. Introduction

The COVID-19 pandemic, a global health crisis, has precipitated unprecedented challenges across healthcare systems, leading to millions of fatalities, economic setbacks, and highlighting disparities in governmental responses worldwide [1]. This crisis promoted an immediate prioritisation of COVID-19 control, including vaccine research and development, and resource allocation for testing and treatment, unintentionally sideling routine health services [2,3]. The diversion of attention and resources has severely disrupted essential healthcare services, particularly in the realms of screening, diagnosis, and management of non-communicable diseases (NCDs), leading to delayed or missed care [2]. Patients, as a result, often present in more advanced stages of diseases or with severe complications, exacerbated by the lockdown measures [2]. Furthermore, COVID-19 infections have a clear direct impact on individual health beyond the primary infection, increasing morbidity and mortality in cardiovascular, neurological, and endocrinological conditions compared with the non-infected population [4]. This, alongside various social restrictions associated with the pandemic, impacted mental health, health behaviours, and social life, including vaccination uptake and, consequently, the burden associated with non-COVID-19 diseases [5,6].

In the case of Japan, the population demonstrated high compliance with non-mandatory lockdown orders and vaccination campaigns, with about 74.6% of the total population having received two doses of the vaccine by the end of 2021 [7,8,9]. Additionally, Japan’s non-centralized healthcare structure for managing the pandemic presents a unique case study [10]. These factors contribute to a distinct context that warrants investigation.

We hypothesize that widespread COVID-19 transmission and associated disruptions led to shifts in risk factors and patterns of disease, resulting in increased mortality from specific causes that are identifiable at the population level. To test this hypothesis, we conducted a comprehensive analysis of the major causes of death in Japan using vital registration data. This study examines changes in mortality trends stratified by cause of death and sex, offering critical insights into the indirect health impacts of the pandemic and informing future public health strategies.

## 2. Methods

### 2.1. Data Source

This study employed a retrospective design with a cross-sectional analysis of vital registration data collected before and after the COVID-19 pandemic. Individual mortality records from the Japanese Ministry of Health Labour and Welfare (MHLW) vital registration database from January 2018 to December 2021 were analysed. Each record included date of death, age at death, sex, and primary cause of death, as classified by the International Statistical Classification of Diseases and Related Health Problems, 10th Revision (ICD-10). Causes of deaths directly related to COVID-19 or those with small and unstable death counts were excluded, as detailed in Table 1. We excluded chapters related to eye diseases (ICD-10 Chapter VII, H00-H59), ear diseases (ICD-10 Chapter VIII, H60-H95), and congenital malformations (ICD-10 Chapter XVII, Q00-Q99) due to their relatively low death counts, which could compromise modeling reliability. ICD-10 Chapter XXI (Z00–Z99), covering factors influencing health status and contact with health services, was not present in our dataset and thus excluded. Additionally, we omitted the special causes of death chapter (ICD-10 Chapter XX, V01-Y98) as it contains COVID-19-related mortality, which was beyond our study’s scope.

To ensure stable estimates, we constrained our analysis to individuals aged 25 to 85 years and over, categorized in five-year age groups, as younger age groups exhibited very low mortality rates that could challenge modeling accuracy. The time period from 2018 to 2021 was chosen so that the study covered two years before the start of the pandemic and two years after. The commencement of the COVID-19 pandemic was marked as January 2020, corresponding with the month when the first case of infection was identified in Japan. Mortality rates were calculated using the population in July of each year [11].

### 2.2. Statistical Analysis

Deaths were counted by month, age group (in 5-year intervals starting from 25 years old), sex, and cause of death groups. The analysis excluded younger age groups due to their very low mortality rates. For each ICD-10 chapter and sex, we fitted separate Poisson regression models with monthly death counts as the outcome variable. The logarithm of the corresponding age–sex group population was included as an offset term to account for population size differences.

The models included three primary predictors: age group (categorical), time (monthly increments centered at January 2020), and a COVID-19 indicator (binary: 0 for pre-January 2020, 1 for subsequent months). To comprehensively capture mortality patterns, we incorporated interaction terms between these predictors. The age-group–time interaction allowed for varying mortality trends across age groups, while the age- group–COVID-19 interaction captured pandemic-specific changes in mortality levels by age. The time–COVID-19 interaction measured changes in overall mortality trends during the pandemic. A three-way interaction between all predictors enabled the detection of age-specific changes in mortality trends during the pandemic period. No other covariates were included in the analysis.

Following the initial model fitting, backward stepwise elimination was used to achieve the most parsimonious model while retaining essential variables. The results are presented as incidence rate ratios (IRRs) with 95% confidence intervals (CIs), calculated from the exponentiated model coefficients. All of the analyses were conducted using R version 4.3.3.

## 3. Results

In the chapters included in our analysis, a total of 4,920,942 deaths from eligible causes were recorded among adults aged 25 and over during the four-year study period. Of these, 2,456,750 deaths occurred during the pandemic years (2020–2021). The leading causes of mortality in 2020–2021 were cancer (791,581 deaths), circulatory diseases (706,795 deaths), and respiratory diseases (349,653 deaths).

**Figure 1:**
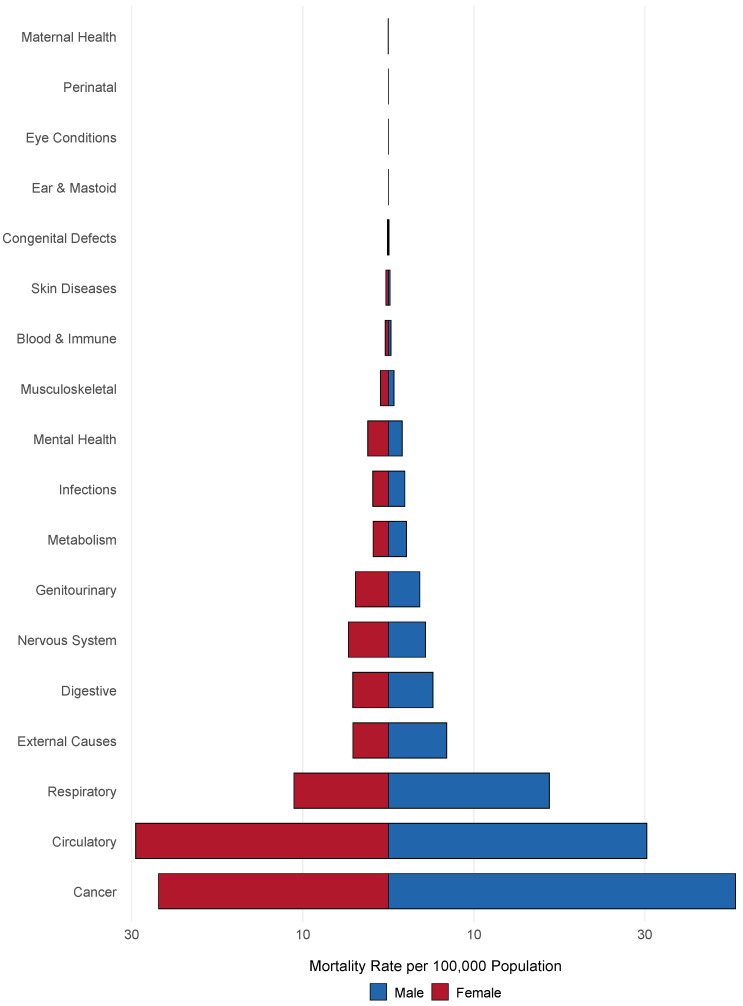
Mortality distribution per 100,000 by cause and sex, 2020–2021.

Figure 1 highlights the top causes of death per 100,000 individuals by sex. The leading causes of death for both men and women were cancer, circulatory diseases, and respiratory diseases. Mortality rates for cancer were higher in men (40.59 per 100,000) than in women (26.87 per 100,000), as were rates for respiratory diseases (18.83 in men vs. 11.05 in women). Circulatory diseases showed comparable rates between sexes (30.22 in men vs. 29.54 in women). Notable differences were observed in external causes, with men having a higher mortality rate (6.82 per 100,000) than women (4.15 per 100,000), and mental health disorders, where women had a higher rate (2.42 per 100,000) than men (1.63 per 100,000).

### 3.1. Mortality Modelling

**Figure 2:**
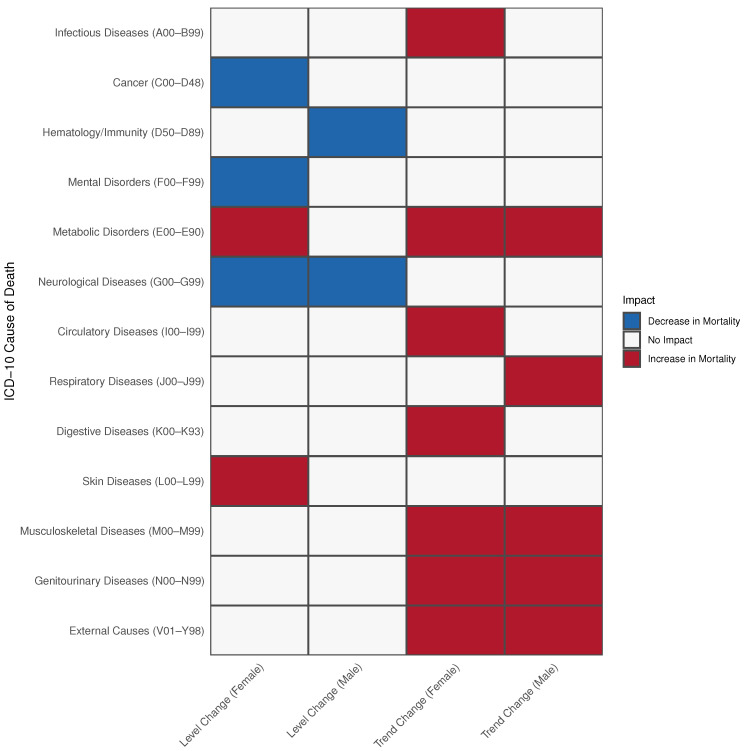
Summary of COVID-19 pandemic impact on mortality trends and levels by cause of death, sex, and age group using the Poisson model. Level changes represent immediate shifts in mortality following the pandemic onset (January 2020), where coefficients greater than 1 indicate an acute increase in average mortality, while coefficients below 1 indicate an acute decrease. Trend changes capture the long-term trajectory of mortality over time during the pandemic period, where interaction coefficients greater than 1 indicate an accelerating upward trend, and coefficients below 1 indicate a declining trend compared with pre-pandemic patterns. Level changes were assessed using the pandemic term coefficient, while trend changes were determined by the interaction between pandemic and time variables.

Figure 2 summarizes the results of the Poisson regression model. No interactions between age groups and the pandemic were observed, indicating no specific age group was uniquely affected by the pandemic. In the following sections, we highlight selected chapters that exhibited substantial changes. Detailed regression tables for all models, including those showing the pandemic’s impact, are provided in the Supplementary Materials.

### 3.2. Diseases of the Endocrine System (E00–E89)

**Figure 3:**
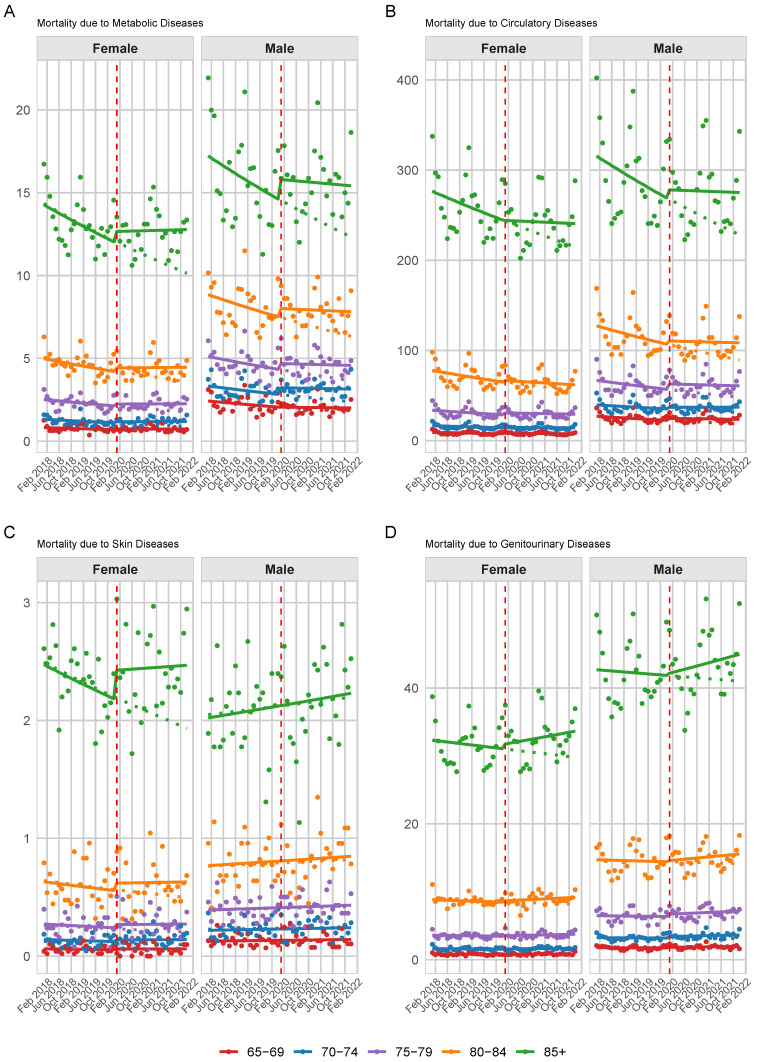
Predicted (Solid line) vs. actual death (dots) rates for: (**A**) metabolic disease mortality (E00–E90), (**B**) circulatory disease mortality (I00–I99), (**C**) skin disease mortality (L00–L99), and (**D**) genitourinary disease mortality (N00–N99) by age group and gender. The vertical dashed line marks the onset of the COVID-19 pandemic and the dotted lines represent the pre-pandemic trend. Note: The scale of the Y-axis varies between plots.

The pandemic was associated with a significant increase in average female mortality due to endocrine-related causes (IRR: 1.058 [1.019, 1.099]), without significant age-specific changes, indicating a uniform impact across age groups. There was a minor significant change in long-term mortality trends post-pandemic for both sexes (Male IRR: 1.006 [1.003, 1.009], Female IRR: 1.008 [1.005, 1.011]). After backward selection, the interaction between age group and the pandemic was retained in the male model; however, it was insignificant for all age groups. Figure 3A shows the predicted trend with observed monthly rates for the five age groups with the highest mortality rates, separately by sex.

### 3.3. Diseases of the Circulatory System (I00–I99)

During the pandemic, the increase in average mortality rates was not statistically significant for both men (IRR: 1.354 [0.981, 1.876]) and women (IRR: 1.157 [0.675, 1.985]). In terms of long-term mortality trends, men showed a non-significant change (IRR: 1.000 [0.977, 1.023]). In contrast, women exhibited a significant increase in long-term mortality (IRR: 1.005 [1.004, 1.005]), indicating a reversal of the pre-pandemic downward trend in circulatory mortality. Regarding the interaction of the pandemic with age groups, both men’s and women’s models retained the interaction, although it was insignificant. However, the men’s model only retained the three-way interaction (age group, time, and pandemic) which was also insignificant. The predicted trend with observed monthly rates for the five age groups with the highest mortality rates, separately by sex, are shown in Figure 3B.

### 3.4. Diseases of the Skin and Subcutaneous Tissue (L00–L99)

For women, there was an increase in mortality levels due to COVID-19 (1.116 [1.014, 1.229]), pointing towards a gender-specific impact of the pandemic. The time interaction with COVID-19 did not reveal a significant change in the trend of mortality rates over the pandemic period for men. For women, however, there was a slight nonsignificant change (1.006 [0.999, 1.013]). The predicted trend with observed monthly rates for the five age groups with the highest mortality rates, separately by sex, are shown in Figure 3C.

### 3.5. Diseases of the Genitourinary System (N00–N99)

The change in mortality trends due to the pandemic, as indicated by the time and COVID-19 interaction, shows a small but statistically significant upward shift for both men (IRR: 1.004 [1.002, 1.006]) and women (IRR: 1.004 [1.002, 1.006]), pointing to a positive change in the trend of mortality rates as the pandemic progressed. The pandemic interaction was retained only in the male model across different age groups; however, it was insignificant. The predicted trend with observed monthly rates for the five age groups with the highest mortality rates, separately by sex, are shown in Figure 3D.

## 4. Discussion

This work provides a comprehensive analysis of the extensive impacts of the COVID-19 pandemic on the Japanese population. We used a Poisson regression model to assess changes in mortality rates across various causes, finding small but significant rises in mortality levels and trends within ICD-10 chapters associated with non-communicable diseases. Specifically, metabolic disorders have exhibited a significant increase in mortality rates for both genders. In the case of circulatory diseases, which are also linked to non-communicable diseases, we observed a shift in the long-term mortality trend exclusively in women. Diseases pertaining to the digestive, musculoskeletal, and genitourinary systems demonstrated an elevation in the long-term mortality trend during the pandemic for both genders. Mortality from infectious diseases showed an increased trend only in women. An unexpected observation was the acute rise in mortality due to skin disorders among women.

This significant increase in mortality associated with chronic diseases included some chapters associated with common disorders that have a significant associated disease burden. We found increases in the metabolic chapter, which contains diseases such as diabetes, and the cardiovascular chapter, which contains diseases like hypertension. Effective management of these conditions relies heavily on early detection and consistent medical intervention to prevent acute complications like malignant hypertension or diabetic ketoacidosis [12]. This management necessitates regular physician visits for monitoring and medication refills. However, during the pandemic, healthcare resources were diverted from chronic disease management to focus on acute COVID-19 care, and many patients avoided medical visits due to fear of infection [2,13]. For example, a report from the USA showed an increase in the incidence of hyperglycemic crises during the pandemic, a direct outcome of lack of access to diabetes healthcare [2,14,15]. In Japan, public health centers (PHCs) play a pivotal role in managing elderly health and non-communicable diseases [16]. Their reallocation to COVID-19 efforts likely helped control the virus’s spread and improve patient outcomes, but also strained healthcare workers and reduced the attention given to chronic disease care [10]. This strain may explain the pandemic’s disproportionate impact on chronic disease mortality among women in Japan. Moreover, there is clear evidence that long-term sequelae of COVID-19 infection worsen the outcomes of many chronic diseases, especially cardiovascular and metabolic conditions [4]. While COVID-19 vaccination could mitigate some of these negative impacts, reports indicate that women exhibited higher vaccine hesitancy compared to men, potentially increasing their vulnerability [17]. These factors underscore the complex interplay between the pandemic and chronic disease outcomes, particularly among women [18,19].

We also identified a substantial rise in mortality due to genitourinary causes, especially pronounced among women. Research in Italy found a decrease in hospital visits for febrile urinary tract infections, primarily due to fears of COVID-19 exposure [20]. This reluctance to seek timely medical care often led to complications, such as sepsis, which, in some cases, resulted in fatalities. The triggering effect of COVID-19 on kidney disease, as evidenced in our study, may have further compounded these risks [21]. This scenario underscores the pandemic’s negative impact on genitourinary health, highlighting an unexpected change in public health risk during such global health crises.

Our study has revealed a surprising finding: a significant increase in mortality due to skin disorders, exclusively among women. Although this change in trend might reflect the direct dermatological impacts of COVID-19, as suggested by previous research [22], in the context of Japan’s ageing society, it could be linked to delayed care for complications like bed ulcers, potentially exacerbated during the pandemic due to limited access to healthcare [23]. Moreover, a concerning factor that might have amplified this trend is the rise in antibiotic-resistant infections [24]. Another critical aspect to consider is the immune modulatory effect of COVID-19, as reports suggest that COVID-19 itself may cause flares of bullous skin diseases, or even the COVID-19 vaccination may have initiated these diseases [25,26].

A majority of the ICD-10 cause categories did not exhibit significant changes in mortality trends and some saw small reductions in excess mortality, a finding that merits attention in the context of Japan’s pandemic response. This relative stability across numerous health categories can be primarily attributed to the country’s proactive promotion of the voluntary lockdowns controlling the infection rates in the years 2020 and 2021, and early implementation of vaccination programs. The country began its vaccination program early, in February 2021, and by early October 2021, it had already achieved approximately 50% coverage of the second dose of the vaccine. This proactive approach ensured that a significant portion of the population was vaccinated before the highest peak of COVID-19 cases, which occurred in late December 2022 [9,27], as evidence showed that vaccination can lower the risk of developing post-COVID-19 infection sequalae [18,28].

### Limitations

Our study has five key limitations. Most critically, we had access only to underlying causes of death, without secondary causes, which may underestimate COVID-19’s true impact as it could have contributed to deaths while not being recorded as the primary cause. Additionally, our use of broad ICD-10 chapters rather than specific disease codes might mask important disease-specific mortality patterns. The study’s reliance on five-year age groups, particularly for those aged 85 and above, may obscure finer mortality patterns in Japan’s aging population. Our focus on adult populations excluded children and adolescents, although mortality in these groups is comparatively rare. Finally, our analysis period ending in 2021 prevents examination of longer-term effects, including potential mortality displacement where increased deaths during the pandemic might lead to decreased mortality in subsequent years. Future research using more granular disease classifications, secondary causes of death, and extended follow-up periods would address these limitations.

## 5. Conclusions

Our study offers pivotal insights into the COVID-19 pandemic’s impact on mortality trends in Japan, particularly highlighting increases in non-communicable diseases and unveiling unique gender-specific effects. Crucially, it underscores the need for targeted healthcare interventions and policy adaptations responsive to such unprecedented health crises. This research not only enhances our understanding of the pandemic’s multifaceted effects, but also serves as a vital guide for future public health strategies and actions, both in Japan and internationally.

## Figures and Tables

**Table 1:** Disease groups used in the study and their corresponding ICD-10 codes.

Chapter	Block	Title	Included	Abbreviated Name
I	A00–B99	Certain infectious and parasitic diseases	Yes	Infectious Diseases
II	C00–D48	Neoplasms	Yes	Cancer
III	D50–D89	Diseases of the blood and blood-forming organs and certain disorders involving the immune mechanism	Yes	Hematology/Immunity
IV	E00–E90	Endocrine, nutritional and metabolic diseases	Yes	Metabolic Disorders
V	F00–F99	Mental and behavioural disorders	Yes	Mental Disorders
VI	G00–G99	Diseases of the nervous system	Yes	Neurological Diseases
VII	H00–H59	Diseases of the eye and adnexa	No	—
VIII	H60–H95	Diseases of the ear and mastoid process	No	—
IX	I00–I99	Diseases of the circulatory system	Yes	Circulatory Diseases
X	J00–J99	Diseases of the respiratory system	Yes	Respiratory Diseases
XI	K00–K93	Diseases of the digestive system	Yes	Digestive Diseases
XII	L00–L99	Diseases of the skin and subcutaneous tissue	Yes	Skin Diseases
XIII	M00–M99	Diseases of the musculoskeletal system and connective tissue	Yes	Musculoskeletal Diseases
XIV	N00–N99	Diseases of the genitourinary system	Yes	Genitourinary Diseases
XV	O00–O99	Pregnancy, childbirth and the puerperium	Yes	Maternal Health
XVI	P00–P96	Certain conditions originating in the perinatal period	No	—
XVII	Q00–Q99	Congenital malformations, deformations and chromosomal abnormalities	Yes	Congenital Defects
XVIII	R00–R99	Symptoms, signs and abnormal clinical and laboratory findings, not elsewhere classified	No	Clinical Findings
XIX	S00–T98	Injury, poisoning and certain other consequences of external causes	Yes	External Causes
XX	V01–Y98	External causes of morbidity and mortality	Yes	External Causes
XXI	Z00–Z99	Factors influencing health status and contact with health services	No	—
XXII	U00–U99	Codes for special purposes	No	—

## Data Availability

The mortality data have been obtained through a restricted data-use agreement with the Ministry of Health, Labour, and Welfare, Japan, and are therefore not available for public dissemination.

## Supplementary Materials

The following supporting information can be downloaded at: https://www.mdpi.com/article/10.3390/ijerph22010074/s1; Supplementary File S1: Full Modeling Results for “The Unseen Aftermath: Effects of COVID-19 Pandemic on Mortality Trends in Japan”.

## Abbreviations

COVID-19	Coronavirus Disease of 2019
ICD-10	International Classification of Diseases, 10th Revision
MHLW	Ministry of Health Labour and Welfare
NCDs	Non-Communicable Diseases
PHCs	Public Health Centers
IRR	Incidence Rate Ratio
CI	Confidence Interval
