# Supplementary material for: The Unseen Aftermath: Associations Between the COVID-19 Pandemic and Shifts in Mortality Trends in Japan
Source: Int J Environ Res Public Health. 2025 Jan 8;22(1):74. doi: 10.3390/ijerph22010074 (PMC11765402; doi:10.3390/ijerph22010074)
Supplement: Supplementary file 1 [file ijerph-22-00074-s001.zip › ijerph-3345748-supplementary.pdf]

Full Modeling Results for "The Unseen Aftermath: Associations Between the COVID-19 Pandemic and Shifts in Mortality Trends in Japan"

A00–B99 Certain infectious and parasitic diseases

	Female		Male	
	Estimate (95% CI)	P-Value[a]	Estimate (95% CI)	P-Value[a]
Yearly Trend				
Time	0.994 [0.992, 0.996]	<0.001	0.987 [0.933, 1.043]	0.639
Age Group				
30-34	1.773 [1.104, 2.847]	0.018	1.477 [0.506, 4.312]	0.475
35-39	1.921 [1.214, 3.041]	0.005	2.305 [0.872, 6.092]	0.092
40-44	2.78 [1.807, 4.276]	<0.001	2.454 [0.967, 6.224]	0.059
45-49	4.015 [2.655, 6.071]	<0.001	5.008 [2.097, 11.962]	<0.001
50-54	8.116 [5.436, 12.118]	<0.001	10.183 [4.346, 23.859]	<0.001
55-59	13.055 [8.788, 19.393]	<0.001	17.354 [7.479, 40.267]	<0.001
60-64	23.221 [15.707, 34.329]	<0.001	29.934 [12.991, 68.973]	<0.001
65-69	44.151 [29.973, 65.035]	<0.001	57.853 [25.267, 132.463]	<0.001
70-74	76.993 [52.337, 113.263]	<0.001	86.502 [37.841, 197.737]	<0.001
75-79	156.536 [106.478, 230.127]	<0.001	144.113 [63.111, 329.08]	<0.001
80-84	315.227 [214.504, 463.246]	<0.001	292.404 [128.147, 667.203]	<0.001
85+	756.327 [514.866, 1111.027]	<0.001	682.832 [299.57, 1556.43]	<0.001
Pandemic				
Pandemic	0.972 [0.938, 1.008]	0.126	1.036 [0.338, 3.173]	0.951
Yearly Trend and Pandemic Interaction				
Time * Pandemic	1.004 [1.001, 1.006]	0.006	1.027 [0.95, 1.11]	0.505
Age and Time Interaction				
30-34 * Time	NI	NI	1.017 [0.945, 1.095]	0.654
35-39 * Time	NI	NI	1.014 [0.949, 1.083]	0.686
40-44 * Time	NI	NI	0.994 [0.934, 1.059]	0.86
45-49 * Time	NI	NI	0.988 [0.932, 1.048]	0.689
50-54 * Time	NI	NI	0.994 [0.938, 1.053]	0.839
55-59 * Time	NI	NI	0.995 [0.94, 1.053]	0.857
60-64 * Time	NI	NI	1.003 [0.948, 1.062]	0.909
65-69 * Time	NI	NI	1.011 [0.956, 1.069]	0.707
70-74 * Time	NI	NI	1.008 [0.953, 1.066]	0.787
75-79 * Time	NI	NI	1.004 [0.949, 1.062]	0.894
80-84 * Time	NI	NI	1.01 [0.955, 1.068]	0.731
85+ * Time	NI	NI	1.01 [0.956, 1.068]	0.716
Age and Pandemic Interaction				
30-34 * Pandemic	NI	NI	1.816 [0.437, 7.549]	0.412

**35-39 * Pandemic**	NI	NI	0.716 [0.185, 2.764]	0.628
**40-44 * Pandemic**	NI	NI	1.938 [0.556, 6.754]	0.299
**45-49 * Pandemic**	NI	NI	1.143 [0.349, 3.738]	0.825
**50-54 * Pandemic**	NI	NI	0.994 [0.312, 3.171]	0.992
**55-59 * Pandemic**	NI	NI	1.033 [0.328, 3.249]	0.956
**60-64 * Pandemic**	NI	NI	1.021 [0.328, 3.179]	0.972
**65-69 * Pandemic**	NI	NI	0.963 [0.312, 2.975]	0.948
**70-74 * Pandemic**	NI	NI	0.937 [0.304, 2.887]	0.91
**75-79 * Pandemic**	NI	NI	1.007 [0.328, 3.097]	0.99
**80-84 * Pandemic**	NI	NI	0.982 [0.32, 3.017]	0.975
**85+ * Pandemic**	NI	NI	0.893 [0.291, 2.74]	0.844
**Three-Way Interaction**				
**30-34 * Time * Pandemic**	NI	NI	0.907 [0.816, 1.008]	0.071
**35-39 * Time * Pandemic**	NI	NI	0.985 [0.896, 1.083]	0.751
**40-44 * Time * Pandemic**	NI	NI	0.962 [0.881, 1.05]	0.385
**45-49 * Time * Pandemic**	NI	NI	1 [0.92, 1.086]	0.993
**50-54 * Time * Pandemic**	NI	NI	0.998 [0.921, 1.082]	0.97
**55-59 * Time * Pandemic**	NI	NI	0.989 [0.913, 1.071]	0.786
**60-64 * Time * Pandemic**	NI	NI	0.978 [0.903, 1.059]	0.579
**65-69 * Time * Pandemic**	NI	NI	0.966 [0.893, 1.045]	0.393
**70-74 * Time * Pandemic**	NI	NI	0.98 [0.907, 1.06]	0.622
**75-79 * Time * Pandemic**	NI	NI	0.986 [0.911, 1.066]	0.716
**80-84 * Time * Pandemic**	NI	NI	0.972 [0.899, 1.051]	0.474
**85+ * Time * Pandemic**	NI	NI	0.973 [0.9, 1.052]	0.495
NI: Not Included (the term was dropped during the backward selection)				
[a] P-values less than 0.001 are reported as '<0.001'.				

C00–D48 Neoplasms

	Female		Male	
	Estimate (95% CI)	P-Value[a]	Estimate (95% CI)	P-Value[a]
Yearly Trend				
Time	0.998 [0.992, 1.004]	0.538	0.998 [0.987, 1.01]	0.794
Age Group				
30-34	2.384 [2.145, 2.649]	<0.001	1.412 [1.112, 1.792]	0.005
35-39	4.481 [4.065, 4.94]	<0.001	2.757 [2.223, 3.42]	<0.001
40-44	8.327 [7.585, 9.141]	<0.001	4.645 [3.795, 5.684]	<0.001
45-49	14.206 [12.964, 15.568]	<0.001	9.741 [8.021, 11.831]	<0.001
50-54	23.733 [21.671, 25.99]	<0.001	19.142 [15.803, 23.188]	<0.001
55-59	35.515 [32.441, 38.879]	<0.001	38.899 [32.164, 47.044]	<0.001
60-64	49.233 [44.984, 53.883]	<0.001	73.823 [61.098, 89.199]	<0.001
65-69	72.23 [66.016, 79.028]	<0.001	129.535 [107.266, 156.428]	<0.001
70-74	100.925 [92.256, 110.41]	<0.001	210.651 [174.471, 254.334]	<0.001
75-79	144.953 [132.508, 158.568]	<0.001	303.239 [251.166, 366.109]	<0.001
80-84	217.37 [198.715, 237.776]	<0.001	441.793 [365.927, 533.387]	<0.001
85+	375.661 [343.46, 410.881]	<0.001	699.001 [579.012, 843.854]	<0.001
Pandemic				
Pandemic	0.97 [0.96, 0.979]	<0.001	0.982 [0.707, 1.363]	0.912
Age and Time Interaction				
30-34 * Time	1.002 [0.995, 1.01]	0.572	0.996 [0.981, 1.011]	0.596
35-39 * Time	1 [0.993, 1.007]	0.943	1.003 [0.989, 1.017]	0.685
40-44 * Time	1.001 [0.994, 1.008]	0.831	0.996 [0.984, 1.009]	0.571
45-49 * Time	1 [0.994, 1.007]	0.942	1.001 [0.988, 1.013]	0.91
50-54 * Time	1.002 [0.996, 1.009]	0.519	1 [0.988, 1.012]	0.968
55-59 * Time	1.002 [0.996, 1.009]	0.511	1.001 [0.989, 1.013]	0.915
60-64 * Time	1.001 [0.995, 1.008]	0.676	0.999 [0.987, 1.011]	0.832
65-69 * Time	1.002 [0.995, 1.008]	0.645	0.999 [0.987, 1.011]	0.808
70-74 * Time	1.003 [0.997, 1.01]	0.32	1.003 [0.991, 1.015]	0.642
75-79 * Time	1.003 [0.997, 1.01]	0.312	1.003 [0.991, 1.015]	0.632
80-84 * Time	1.003 [0.996, 1.009]	0.414	1.003 [0.991, 1.015]	0.674
85+ * Time	1.003 [0.996, 1.009]	0.396	1.003 [0.991, 1.015]	0.662
Age and Pandemic Interaction				
30-34 * Pandemic	NI	NI	1.3 [0.86, 1.964]	0.213
35-39 * Pandemic	NI	NI	0.951 [0.653, 1.385]	0.792
40-44 * Pandemic	NI	NI	1.086 [0.764, 1.544]	0.647
45-49 * Pandemic	NI	NI	0.964 [0.687, 1.353]	0.833

**50-54 * Pandemic**	NI	NI	0.994 [0.712, 1.389]	0.972
**55-59 * Pandemic**	NI	NI	0.982 [0.705, 1.368]	0.916
**60-64 * Pandemic**	NI	NI	1.005 [0.722, 1.397]	0.978
**65-69 * Pandemic**	NI	NI	1.041 [0.749, 1.447]	0.81
**70-74 * Pandemic**	NI	NI	0.966 [0.696, 1.342]	0.838
**75-79 * Pandemic**	NI	NI	0.998 [0.719, 1.387]	0.992
**80-84 * Pandemic**	NI	NI	0.965 [0.695, 1.34]	0.832
**85+ * Pandemic**	NI	NI	0.961 [0.692, 1.334]	0.812

NI: Not Included (the term was dropped during the backward selection)

[a] P-values less than 0.001 are reported as '<0.001'.

D50–D89 Diseases of the blood and blood-forming organs and certain disorders involving the immune mechanism

	Female		Male	
	Estimate (95% CI)	P-Value[a]	Estimate (95% CI)	P-Value[a]
**Yearly Trend**				
**Time**	1.015 [0.928, 1.111]	0.745	NI	NI
**Age Group**				
**30-34**	1.042 [0.183, 5.943]	0.963	1.52 [0.761, 3.035]	0.235
**35-39**	1.521 [0.301, 7.685]	0.612	1.761 [0.909, 3.413]	0.094
**40-44**	1.97 [0.435, 8.915]	0.379	2.548 [1.375, 4.723]	0.003
**45-49**	1.848 [0.426, 8.012]	0.412	3.291 [1.814, 5.969]	<0.001
**50-54**	5.636 [1.387, 22.896]	0.016	6.313 [3.553, 11.216]	<0.001
**55-59**	9.844 [2.47, 39.23]	0.001	8.5 [4.808, 15.025]	<0.001
**60-64**	11.345 [2.895, 44.463]	<0.001	16.852 [9.65, 29.427]	<0.001
**65-69**	16.3 [4.245, 62.585]	<0.001	25.312 [14.578, 43.948]	<0.001
**70-74**	22.965 [6.025, 87.533]	<0.001	46.401 [26.823, 80.27]	<0.001
**75-79**	59.09 [15.611, 223.661]	<0.001	85.867 [49.699, 148.357]	<0.001
**80-84**	79.756 [21.101, 301.456]	<0.001	162.577 [94.178, 280.652]	<0.001
**85+**	265.912 [70.656, 1000.756]	<0.001	353.573 [205.045, 609.692]	<0.001
**Pandemic**				
**Pandemic**	0.433 [0.035, 5.339]	0.514	0.945 [0.902, 0.99]	0.016
**Yearly Trend and Pandemic Interaction**				
**Time * Pandemic**	1.006 [1, 1.012]	0.062	NI	NI
**Age and Time Interaction**				
**30-34 * Time**	1.013 [0.903, 1.137]	0.824	NI	NI
**35-39 * Time**	1.003 [0.899, 1.119]	0.959	NI	NI
**40-44 * Time**	0.984 [0.889, 1.089]	0.758	NI	NI
**45-49 * Time**	0.966 [0.876, 1.065]	0.488	NI	NI
**50-54 * Time**	1.019 [0.926, 1.12]	0.704	NI	NI
**55-59 * Time**	1.032 [0.939, 1.134]	0.516	NI	NI
**60-64 * Time**	1.003 [0.915, 1.101]	0.942	NI	NI
**65-69 * Time**	0.981 [0.896, 1.075]	0.688	NI	NI
**70-74 * Time**	0.98 [0.895, 1.073]	0.666	NI	NI
**75-79 * Time**	0.989 [0.903, 1.082]	0.804	NI	NI
**80-84 * Time**	0.976 [0.892, 1.068]	0.593	NI	NI
**85+ * Time**	0.985 [0.901, 1.078]	0.748	NI	NI
**Age and Pandemic Interaction**				
**30-34 * Pandemic**	1.945 [0.078, 48.298]	0.685	NI	NI
**35-39 * Pandemic**	1.333 [0.063, 28.086]	0.853	NI	NI
**40-44 * Pandemic**	1.823 [0.108, 30.837]	0.677	NI	NI
**45-49 * Pandemic**	3.698 [0.242, 56.39]	0.347	NI	NI
**50-54 * Pandemic**	1.186 [0.083, 16.881]	0.9	NI	NI

**55-59 * Pandemic**	0.67 [0.048, 9.33]	0.765	NI	NI
**60-64 * Pandemic**	1.376 [0.103, 18.295]	0.809	NI	NI
**65-69 * Pandemic**	2.233 [0.175, 28.526]	0.537	NI	NI
**70-74 * Pandemic**	2.532 [0.201, 31.94]	0.473	NI	NI
**75-79 * Pandemic**	1.835 [0.147, 22.911]	0.638	NI	NI
**80-84 * Pandemic**	2.896 [0.233, 36.047]	0.409	NI	NI
**85+ * Pandemic**	2.043 [0.165, 25.269]	0.578	NI	NI
NI: Not Included (the term was dropped during the backward selection)				
[a] P-values less than 0.001 are reported as '<0.001'.				

E00–E90 Endocrine, nutritional and metabolic diseases

	Female		Male	
	**Estimate (95% CI)**	**P-Value[a]**	**Estimate (95% CI)**	**P-Value[a]**
**Yearly Trend**				
**Time**	0.993 [0.991, 0.995]	<0.001	0.993 [0.991, 0.995]	<0.001
**Age Group**				
**30-34**	1.477 [1.018, 2.144]	0.04	1.896 [1.177, 3.055]	0.009
**35-39**	2.387 [1.701, 3.349]	<0.001	3.069 [1.973, 4.773]	<0.001
**40-44**	2.887 [2.084, 4.001]	<0.001	5.449 [3.593, 8.264]	<0.001
**45-49**	4.331 [3.167, 5.921]	<0.001	10.226 [6.825, 15.322]	<0.001
**50-54**	7.349 [5.412, 9.98]	<0.001	17.549 [11.759, 26.19]	<0.001
**55-59**	10.036 [7.41, 13.593]	<0.001	28.471 [19.128, 42.378]	<0.001
**60-64**	13.321 [9.86, 17.996]	<0.001	42.101 [28.335, 62.554]	<0.001
**65-69**	24.214 [18.006, 32.564]	<0.001	68.861 [46.435, 102.119]	<0.001
**70-74**	38.965 [29.022, 52.314]	<0.001	94.596 [63.811, 140.233]	<0.001
**75-79**	74.228 [55.336, 99.571]	<0.001	145.087 [97.901, 215.017]	<0.001
**80-84**	146.491 [109.271, 196.39]	<0.001	251.018 [169.421, 371.914]	<0.001
**85+**	419.266 [312.958, 561.685]	<0.001	488.678 [329.965, 723.731]	<0.001
**Pandemic**				
**Pandemic**	1.058 [1.019, 1.099]	0.003	1.654 [0.997, 2.742]	0.051
**Yearly Trend and Pandemic Interaction**				
**Time * Pandemic**	1.008 [1.005, 1.011]	<0.001	1.006 [1.003, 1.009]	<0.001
**Age and Pandemic Interaction**				
**30-34 * Pandemic**	NI	NI	0.851 [0.456, 1.588]	0.612
**35-39 * Pandemic**	NI	NI	0.786 [0.441, 1.399]	0.413
**40-44 * Pandemic**	NI	NI	0.677 [0.393, 1.166]	0.16
**45-49 * Pandemic**	NI	NI	0.781 [0.463, 1.317]	0.354
**50-54 * Pandemic**	NI	NI	0.649 [0.387, 1.089]	0.102
**55-59 * Pandemic**	NI	NI	0.694 [0.416, 1.16]	0.164
**60-64 * Pandemic**	NI	NI	0.678 [0.407, 1.13]	0.136
**65-69 * Pandemic**	NI	NI	0.607 [0.365, 1.01]	0.055
**70-74 * Pandemic**	NI	NI	0.694 [0.418, 1.152]	0.157
**75-79 * Pandemic**	NI	NI	0.657 [0.396, 1.091]	0.105
**80-84 * Pandemic**	NI	NI	0.649 [0.391, 1.077]	0.095
**85+ * Pandemic**	NI	NI	0.658 [0.397, 1.091]	0.104
NI: Not Included (the term was dropped during the backward selection)				
[a] P-values less than 0.001 are reported as '<0.001'.				

F00–F99 Mental and behavioural disorders

	Female		Male	
	Estimate (95% CI)	P-Value[a]	Estimate (95% CI)	P-Value[a]
**Yearly Trend**				
**Time**	1.002 [1.001, 1.004]	<0.001	1.001 [0.998, 1.003]	0.634
**Age Group**				
**30-34**	2.333 [1.372, 3.969]	0.002	8.207 [1.041, 64.709]	0.046
**35-39**	2.676 [1.601, 4.474]	<0.001	18.983 [2.567, 140.403]	0.004
**40-44**	4.486 [2.765, 7.279]	<0.001	28.112 [3.869, 204.25]	0.001
**45-49**	5.127 [3.183, 8.257]	<0.001	55.045 [7.673, 394.88]	<0.001
**50-54**	5.6 [3.475, 9.025]	<0.001	102.404 [14.338, 731.405]	<0.001
**55-59**	7.936 [4.953, 12.715]	<0.001	149.389 [20.953, 1065.123]	<0.001
**60-64**	10.067 [6.31, 16.062]	<0.001	277.082 [38.959, 1970.666]	<0.001
**65-69**	18.406 [11.644, 29.094]	<0.001	426.361 [60.03, 3028.234]	<0.001
**70-74**	36.819 [23.393, 57.949]	<0.001	824.378 [116.148, 5851.131]	<0.001
**75-79**	107.012 [68.147, 168.042]	<0.001	1963.12 [276.716, 13927.086]	<0.001
**80-84**	326.388 [208.055, 512.022]	<0.001	5102.521 [719.401, 36190.816]	<0.001
**85+**	1831.29 [1168.019, 2871.205]	<0.001	16786.402 [2367.121, 119040.491]	<0.001
**Pandemic**				
**Pandemic**	0.909 [0.88, 0.939]	<0.001	9.414 [1.206, 73.482]	0.032
**Yearly Trend and Pandemic Interaction**				
**Time * Pandemic**	NI	NI	1.003 [1, 1.006]	0.085
**Age and Pandemic Interaction**				
**30-34 * Pandemic**	NI	NI	0.154 [0.017, 1.424]	0.099
**35-39 * Pandemic**	NI	NI	0.101 [0.012, 0.857]	0.036
**40-44 * Pandemic**	NI	NI	0.121 [0.015, 0.989]	0.049
**45-49 * Pandemic**	NI	NI	0.129 [0.016, 1.023]	0.053
**50-54 * Pandemic**	NI	NI	0.11 [0.014, 0.866]	0.036
**55-59 * Pandemic**	NI	NI	0.114 [0.014, 0.898]	0.039
**60-64 * Pandemic**	NI	NI	0.111 [0.014, 0.872]	0.037
**65-69 * Pandemic**	NI	NI	0.118 [0.015, 0.92]	0.041
**70-74 * Pandemic**	NI	NI	0.112 [0.014, 0.877]	0.037
**75-79 * Pandemic**	NI	NI	0.111 [0.014, 0.867]	0.036
**80-84 * Pandemic**	NI	NI	0.096 [0.012, 0.752]	0.026
**85+ * Pandemic**	NI	NI	0.099 [0.013, 0.775]	0.028
NI: Not Included (the term was dropped during the backward selection)				
[a] P-values less than 0.001 are reported as '<0.001'.				

G00–G99 Diseases of the nervous system

	Female		Male	
	Estimate (95% CI)	P-Value[a]	Estimate (95% CI)	P-Value[a]
**Yearly Trend**				
**Time**	1.004 [1.002, 1.005]	<0.001	0.984 [0.975, 0.994]	0.001
**Age Group**				
**30-34**	1.106 [0.865, 1.414]	0.42	1.024 [0.85, 1.233]	0.806
**35-39**	1.157 [0.913, 1.467]	0.226	1.139 [0.954, 1.36]	0.15
**40-44**	1.715 [1.382, 2.127]	<0.001	1.543 [1.31, 1.816]	<0.001
**45-49**	2.298 [1.873, 2.82]	<0.001	2.393 [2.056, 2.786]	<0.001
**50-54**	3.786 [3.107, 4.612]	<0.001	3.643 [3.142, 4.223]	<0.001
**55-59**	6.127 [5.054, 7.427]	<0.001	6.238 [5.404, 7.2]	<0.001
**60-64**	10.958 [9.082, 13.22]	<0.001	10.696 [9.297, 12.306]	<0.001
**65-69**	19.182 [15.952, 23.066]	<0.001	20.113 [17.53, 23.077]	<0.001
**70-74**	36.328 [30.258, 43.614]	<0.001	36.253 [31.632, 41.549]	<0.001
**75-79**	75.821 [63.196, 90.969]	<0.001	74.261 [64.826, 85.069]	<0.001
**80-84**	153.811 [128.247, 184.471]	<0.001	143.381 [125.196, 164.207]	<0.001
**85+**	420.958 [351.135, 504.664]	<0.001	269.077 [235.007, 308.086]	<0.001
**Pandemic**				
**Pandemic**	0.922 [0.9, 0.944]	<0.001	0.959 [0.935, 0.984]	0.001
**Yearly Trend and Pandemic Interaction**				
**Time * Pandemic**	1.002 [1, 1.003]	0.079	1.002 [1, 1.003]	0.084
**Age and Time Interaction**				
**30-34 * Time**	NI	NI	1.01 [0.997, 1.024]	0.138
**35-39 * Time**	NI	NI	1.015 [1.002, 1.027]	0.026
**40-44 * Time**	NI	NI	1.019 [1.007, 1.031]	0.002
**45-49 * Time**	NI	NI	1.014 [1.004, 1.026]	0.009
**50-54 * Time**	NI	NI	1.017 [1.006, 1.028]	0.002
**55-59 * Time**	NI	NI	1.013 [1.002, 1.023]	0.016
**60-64 * Time**	NI	NI	1.014 [1.004, 1.025]	0.005
**65-69 * Time**	NI	NI	1.018 [1.009, 1.028]	<0.001
**70-74 * Time**	NI	NI	1.019 [1.009, 1.029]	<0.001
**75-79 * Time**	NI	NI	1.019 [1.009, 1.029]	<0.001
**80-84 * Time**	NI	NI	1.018 [1.009, 1.028]	<0.001
**85+ * Time**	NI	NI	1.018 [1.008, 1.028]	<0.001

NI: Not Included (the term was dropped during the backward selection)

[a] P-values less than 0.001 are reported as '<0.001'.

I00–I99 Diseases of the circulatory system

	Female		Male	
	Estimate (95% CI)	P-Value[a]	Estimate (95% CI)	P-Value[a]
**Yearly Trend**				
**Time**	0.994 [0.975, 1.014]	0.558	0.995 [0.978, 1.013]	0.597
**Age Group**				
**30-34**	2.185 [1.489, 3.206]	<0.001	1.776 [1.31, 2.408]	<0.001
**35-39**	3.216 [2.263, 4.569]	<0.001	3.801 [2.884, 5.01]	<0.001
**40-44**	6.225 [4.47, 8.671]	<0.001	6.819 [5.245, 8.865]	<0.001
**45-49**	11.205 [8.117, 15.467]	<0.001	12.884 [9.972, 16.646]	<0.001
**50-54**	16.875 [12.252, 23.242]	<0.001	22.046 [17.096, 28.429]	<0.001
**55-59**	22.539 [16.382, 31.011]	<0.001	31.787 [24.675, 40.95]	<0.001
**60-64**	36.43 [26.532, 50.02]	<0.001	46.387 [36.044, 59.697]	<0.001
**65-69**	56.859 [41.481, 77.939]	<0.001	68.144 [53.002, 87.611]	<0.001
**70-74**	107.936 [78.816, 147.816]	<0.001	107.032 [83.281, 137.555]	<0.001
**75-79**	217.26 [158.71, 297.411]	<0.001	172.69 [134.399, 221.889]	<0.001
**80-84**	490.386 [358.319, 671.129]	<0.001	324.472 [252.568, 416.847]	<0.001
**85+**	1851.39 [1353.131, 2533.12]	<0.001	816.97 [636.109, 1049.253]	<0.001
**Pandemic**				
**Pandemic**	1.157 [0.675, 1.984]	0.596	1.354 [0.98, 1.872]	0.066
**Yearly Trend and Pandemic Interaction**				
**Time * Pandemic**	1.005 [1.004, 1.005]	<0.001	1 [0.977, 1.023]	0.981
**Age and Time Interaction**				
**30-34 * Time**	1.011 [0.987, 1.036]	0.358	0.99 [0.97, 1.011]	0.349
**35-39 * Time**	0.996 [0.975, 1.018]	0.74	1.001 [0.982, 1.02]	0.91
**40-44 * Time**	1.001 [0.98, 1.022]	0.936	0.996 [0.978, 1.014]	0.627
**45-49 * Time**	0.998 [0.979, 1.019]	0.882	1 [0.982, 1.018]	0.983
**50-54 * Time**	1.001 [0.982, 1.021]	0.891	1.004 [0.986, 1.021]	0.693
**55-59 * Time**	1.001 [0.981, 1.021]	0.948	1.002 [0.985, 1.02]	0.814
**60-64 * Time**	1.002 [0.983, 1.022]	0.816	1 [0.983, 1.018]	0.99
**65-69 * Time**	0.997 [0.978, 1.017]	0.769	0.997 [0.979, 1.014]	0.699
**70-74 * Time**	0.999 [0.98, 1.019]	0.953	1 [0.982, 1.017]	0.963
**75-79 * Time**	0.999 [0.98, 1.019]	0.923	0.998 [0.98, 1.015]	0.793
**80-84 * Time**	0.998 [0.979, 1.017]	0.831	0.997 [0.98, 1.015]	0.746
**85+ * Time**	1.001 [0.981, 1.02]	0.959	0.998 [0.981, 1.015]	0.801
**Age and Pandemic Interaction**				
**30-34 * Pandemic**	0.584 [0.298, 1.144]	0.117	0.909 [0.609, 1.358]	0.642
**35-39 * Pandemic**	0.974 [0.532, 1.783]	0.932	0.786 [0.547, 1.128]	0.192
**40-44 * Pandemic**	0.896 [0.506, 1.586]	0.707	0.929 [0.66, 1.307]	0.673
**45-49 * Pandemic**	0.919 [0.527, 1.6]	0.765	0.841 [0.603, 1.172]	0.307
**50-54 * Pandemic**	0.919 [0.53, 1.595]	0.765	0.765 [0.55, 1.064]	0.112

**55-59 * Pandemic**	0.878 [0.507, 1.52]	0.641	0.803 [0.578, 1.115]	0.189
**60-64 * Pandemic**	0.845 [0.49, 1.459]	0.546	0.797 [0.574, 1.105]	0.173
**65-69 * Pandemic**	0.948 [0.551, 1.632]	0.848	0.816 [0.589, 1.13]	0.221
**70-74 * Pandemic**	0.897 [0.522, 1.542]	0.695	0.778 [0.562, 1.076]	0.129
**75-79 * Pandemic**	0.929 [0.541, 1.594]	0.788	0.821 [0.593, 1.136]	0.234
**80-84 * Pandemic**	0.893 [0.52, 1.532]	0.681	0.768 [0.555, 1.062]	0.111
**85+ * Pandemic**	0.866 [0.505, 1.486]	0.602	0.767 [0.555, 1.061]	0.109
**Three-Way Interaction**				
**30-34 * Time * Pandemic**	NI	NI	1.007 [0.979, 1.037]	0.625
**35-39 * Time * Pandemic**	NI	NI	0.998 [0.972, 1.024]	0.882
**40-44 * Time * Pandemic**	NI	NI	0.996 [0.971, 1.02]	0.724
**45-49 * Time * Pandemic**	NI	NI	0.998 [0.975, 1.023]	0.899
**50-54 * Time * Pandemic**	NI	NI	1.002 [0.978, 1.026]	0.885
**55-59 * Time * Pandemic**	NI	NI	1 [0.977, 1.024]	0.986
**60-64 * Time * Pandemic**	NI	NI	1.002 [0.979, 1.026]	0.86
**65-69 * Time * Pandemic**	NI	NI	1.006 [0.983, 1.03]	0.591
**70-74 * Time * Pandemic**	NI	NI	1.006 [0.982, 1.03]	0.635
**75-79 * Time * Pandemic**	NI	NI	1.006 [0.983, 1.03]	0.629
**80-84 * Time * Pandemic**	NI	NI	1.007 [0.984, 1.031]	0.557
**85+ * Time * Pandemic**	NI	NI	1.007 [0.984, 1.031]	0.572
NI: Not Included (the term was dropped during the backward selection)				
[a] P-values less than 0.001 are reported as '<0.001'.				

J00–J99 Diseases of the respiratory system

	Female		Male	
	Estimate (95% CI)	P-Value[a]	Estimate (95% CI)	P-Value[a]
**Yearly Trend**				
**Time**	1.018 [0.98, 1.058]	0.366	0.976 [0.946, 1.006]	0.112
**Age Group**				
**30-34**	1.154 [0.546, 2.439]	0.708	1.543 [0.799, 2.982]	0.196
**35-39**	0.906 [0.437, 1.875]	0.789	2.908 [1.611, 5.248]	<0.001
**40-44**	2.268 [1.191, 4.317]	0.013	3.854 [2.208, 6.727]	<0.001
**45-49**	3.287 [1.781, 6.067]	<0.001	7.288 [4.278, 12.416]	<0.001
**50-54**	5.123 [2.809, 9.342]	<0.001	17.194 [10.198, 28.989]	<0.001
**55-59**	9.709 [5.383, 17.511]	<0.001	34.471 [20.554, 57.81]	<0.001
**60-64**	17.116 [9.573, 30.604]	<0.001	84.608 [50.64, 141.359]	<0.001
**65-69**	42.972 [24.188, 76.345]	<0.001	180.867 [108.447, 301.648]	<0.001
**70-74**	89.281 [50.355, 158.299]	<0.001	414.619 [248.77, 691.035]	<0.001
**75-79**	217.187 [122.608, 384.723]	<0.001	943.45 [566.204, 1572.044]	<0.001
**80-84**	537.964 [303.818, 952.561]	<0.001	2048.629 [1229.605, 3413.196]	<0.001
**85+**	2168.254 [1224.954, 3837.96]	<0.001	6027.565 [3618.258, 10041.167]	<0.001
**Pandemic**				
**Pandemic**	0.53 [0.183, 1.532]	0.241	1.671 [0.717, 3.896]	0.235
**Yearly Trend and Pandemic Interaction**				
**Time * Pandemic**	1.002 [1.001, 1.003]	<0.001	1.004 [1.003, 1.005]	<0.001
**Age and Time Interaction**				
**30-34 * Time**	0.977 [0.93, 1.027]	0.365	1.009 [0.97, 1.05]	0.655
**35-39 * Time**	0.947 [0.904, 0.993]	0.024	1.018 [0.982, 1.055]	0.336
**40-44 * Time**	0.978 [0.937, 1.021]	0.304	1.003 [0.97, 1.038]	0.841
**45-49 * Time**	0.972 [0.933, 1.012]	0.17	1.002 [0.971, 1.035]	0.896
**50-54 * Time**	0.97 [0.932, 1.009]	0.133	1.012 [0.98, 1.044]	0.467
**55-59 * Time**	0.973 [0.935, 1.012]	0.17	1.012 [0.981, 1.044]	0.456
**60-64 * Time**	0.965 [0.928, 1.003]	0.074	1.015 [0.984, 1.047]	0.344
**65-69 * Time**	0.977 [0.94, 1.016]	0.239	1.014 [0.984, 1.046]	0.365
**70-74 * Time**	0.978 [0.942, 1.017]	0.267	1.02 [0.989, 1.052]	0.201
**75-79 * Time**	0.976 [0.939, 1.014]	0.21	1.021 [0.99, 1.053]	0.182
**80-84 * Time**	0.977 [0.94, 1.015]	0.232	1.019 [0.988, 1.051]	0.227
**85+ * Time**	0.976 [0.939, 1.014]	0.209	1.019 [0.989, 1.051]	0.221
**Age and Pandemic Interaction**				
**30-34 * Pandemic**	1.342 [0.34, 5.302]	0.674	0.758 [0.251, 2.288]	0.623
**35-39 * Pandemic**	3.094 [0.844, 11.337]	0.088	0.629 [0.233, 1.694]	0.359
**40-44 * Pandemic**	1.535 [0.469, 5.027]	0.479	0.985 [0.391, 2.483]	0.974

**45-49 * Pandemic**	2.072 [0.67, 6.408]	0.206	1.002 [0.414, 2.424]	0.997
**50-54 * Pandemic**	2.248 [0.742, 6.815]	0.152	0.74 [0.311, 1.762]	0.496
**55-59 * Pandemic**	1.814 [0.608, 5.408]	0.285	0.74 [0.314, 1.747]	0.493
**60-64 * Pandemic**	2.271 [0.773, 6.67]	0.136	0.648 [0.277, 1.52]	0.319
**65-69 * Pandemic**	1.76 [0.605, 5.121]	0.299	0.669 [0.286, 1.562]	0.353
**70-74 * Pandemic**	1.751 [0.604, 5.078]	0.302	0.594 [0.255, 1.387]	0.229
**75-79 * Pandemic**	1.924 [0.664, 5.569]	0.228	0.589 [0.253, 1.375]	0.221
**80-84 * Pandemic**	1.797 [0.621, 5.198]	0.28	0.588 [0.252, 1.371]	0.219
**85+ * Pandemic**	1.727 [0.597, 4.992]	0.313	0.557 [0.239, 1.299]	0.176
NI: Not Included (the term was dropped during the backward selection)				
[a] P-values less than 0.001 are reported as '<0.001'.				

K00–K93 Diseases of the digestive system

	Female		Male	
	Estimate (95% CI)	P-Value[a]	Estimate (95% CI)	P-Value[a]
**Yearly Trend**				
**Time**	1.011 [0.994, 1.029]	0.208	0.998 [0.997, 0.999]	0.003
**Age Group**				
**30-34**	1.758 [1.304, 2.37]	<0.001	2.222 [1.588, 3.11]	<0.001
**35-39**	3.269 [2.494, 4.284]	<0.001	5.261 [3.881, 7.132]	<0.001
**40-44**	6.278 [4.863, 8.105]	<0.001	10.89 [8.133, 14.582]	<0.001
**45-49**	9.136 [7.113, 11.734]	<0.001	19.852 [14.891, 26.466]	<0.001
**50-54**	13.338 [10.404, 17.099]	<0.001	34.758 [26.113, 46.265]	<0.001
**55-59**	17.472 [13.642, 22.377]	<0.001	50.9 [38.269, 67.7]	<0.001
**60-64**	24.454 [19.121, 31.274]	<0.001	65.792 [49.49, 87.464]	<0.001
**65-69**	37.525 [29.392, 47.909]	<0.001	85.448 [64.316, 113.522]	<0.001
**70-74**	58.626 [45.959, 74.785]	<0.001	108.375 [81.584, 143.964]	<0.001
**75-79**	108.463 [85.066, 138.294]	<0.001	165.593 [124.681, 219.93]	<0.001
**80-84**	218.72 [171.598, 278.78]	<0.001	269.818 [203.178, 358.316]	<0.001
**85+**	666.19 [522.871, 848.792]	<0.001	586.261 [441.612, 778.289]	<0.001
**Pandemic**				
**Pandemic**	1.007 [0.982, 1.032]	0.581	1.164 [0.791, 1.713]	0.44
**Yearly Trend and Pandemic Interaction**				
**Time * Pandemic**	1.004 [1.002, 1.005]	<0.001	1.002 [1.001, 1.004]	0.004
**Age and Time Interaction**				
**30-34 * Time**	0.989 [0.968, 1.01]	0.304	NI	NI
**35-39 * Time**	0.994 [0.975, 1.014]	0.553	NI	NI
**40-44 * Time**	0.997 [0.979, 1.015]	0.743	NI	NI
**45-49 * Time**	0.989 [0.971, 1.007]	0.209	NI	NI
**50-54 * Time**	0.994 [0.976, 1.012]	0.503	NI	NI
**55-59 * Time**	0.987 [0.97, 1.005]	0.161	NI	NI
**60-64 * Time**	0.988 [0.971, 1.006]	0.178	NI	NI
**65-69 * Time**	0.987 [0.97, 1.005]	0.16	NI	NI
**70-74 * Time**	0.987 [0.97, 1.004]	0.14	NI	NI
**75-79 * Time**	0.987 [0.97, 1.004]	0.142	NI	NI
**80-84 * Time**	0.986 [0.968, 1.003]	0.102	NI	NI
**85+ * Time**	0.986 [0.969, 1.003]	0.108	NI	NI
**Age and Pandemic Interaction**				
**30-34 * Pandemic**	NI	NI	0.97 [0.612, 1.537]	0.896
**35-39 * Pandemic**	NI	NI	1.001 [0.661, 1.517]	0.995
**40-44 * Pandemic**	NI	NI	0.997 [0.669, 1.485]	0.987
**45-49 * Pandemic**	NI	NI	0.982 [0.664, 1.453]	0.928
**50-54 * Pandemic**	NI	NI	0.926 [0.627, 1.368]	0.699

**55-59 * Pandemic**	NI	NI	0.9 [0.61, 1.328]	0.596
**60-64 * Pandemic**	NI	NI	0.919 [0.623, 1.354]	0.669
**65-69 * Pandemic**	NI	NI	0.892 [0.606, 1.314]	0.564
**70-74 * Pandemic**	NI	NI	0.883 [0.6, 1.301]	0.53
**75-79 * Pandemic**	NI	NI	0.864 [0.587, 1.272]	0.458
**80-84 * Pandemic**	NI	NI	0.871 [0.592, 1.282]	0.485
**85+ * Pandemic**	NI	NI	0.871 [0.592, 1.281]	0.483
NI: Not Included (the term was dropped during the backward selection)				
[a] P-values less than 0.001 are reported as '<0.001'.				

L00–L99 Diseases of the skin and subcutaneous tissue

	Female		Male	
	Estimate (95% CI)	P-Value[a]	Estimate (95% CI)	P-Value[a]
**Yearly Trend**				
**Time**	0.995 [0.99, 1]	0.034	1.002 [1, 1.004]	0.05
**Age Group**				
**30-34**	2.774 [0.56, 13.742]	0.212	0.707 [0.158, 3.158]	0.65
**35-39**	3.283 [0.697, 15.459]	0.133	1.911 [0.589, 6.204]	0.281
**40-44**	3.201 [0.692, 14.815]	0.137	4.245 [1.468, 12.271]	0.008
**45-49**	4.112 [0.928, 18.223]	0.063	8.23 [2.973, 22.781]	<0.001
**50-54**	10.634 [2.541, 44.5]	0.001	13.041 [4.763, 35.711]	<0.001
**55-59**	16.488 [3.991, 68.113]	<0.001	17.786 [6.527, 48.469]	<0.001
**60-64**	23.625 [5.772, 96.691]	<0.001	38.668 [14.358, 104.141]	<0.001
**65-69**	43.629 [10.794, 176.345]	<0.001	52.103 [19.419, 139.795]	<0.001
**70-74**	98.792 [24.595, 396.821]	<0.001	89.155 [33.329, 238.493]	<0.001
**75-79**	191.986 [47.878, 769.85]	<0.001	159.587 [59.721, 426.448]	<0.001
**80-84**	444.517 [111.004, 1780.079]	<0.001	313.095 [117.275, 835.886]	<0.001
**85+**	1742.222 [435.587, 6968.382]	<0.001	824.076 [309.058, 2197.33]	<0.001
**Pandemic**				
**Pandemic**	1.116 [1.014, 1.229]	0.025	NI	NI
**Yearly Trend and Pandemic Interaction**				
**Time * Pandemic**	1.006 [0.999, 1.013]	0.08	NI	NI
NI: Not Included (the term was dropped during the backward selection)				
[a] P-values less than 0.001 are reported as '<0.001'.				

M00–M99 Diseases of the musculoskeletal system and connective tissue

	Female		Male	
	Estimate (95% CI)	P-Value[a]	Estimate (95% CI)	P-Value[a]
Yearly Trend				
**Time**	0.998 [0.995, 1.001]	0.168	1 [0.996, 1.003]	0.845
Age Group				
**30-34**	1.782 [0.935, 3.398]	0.079	0.942 [0.408, 2.172]	0.888
**35-39**	2.754 [1.517, 5.002]	0.001	1.235 [0.573, 2.66]	0.591
**40-44**	4.722 [2.693, 8.283]	<0.001	3.888 [2.041, 7.407]	<0.001
**45-49**	6.869 [3.973, 11.874]	<0.001	7.781 [4.205, 14.398]	<0.001
**50-54**	10.434 [6.072, 17.928]	<0.001	13.758 [7.5, 25.238]	<0.001
**55-59**	17.163 [10.045, 29.324]	<0.001	24.58 [13.478, 44.827]	<0.001
**60-64**	29.738 [17.49, 50.563]	<0.001	44.465 [24.484, 80.752]	<0.001
**65-69**	58.042 [34.271, 98.3]	<0.001	75.654 [41.773, 137.016]	<0.001
**70-74**	94.556 [55.903, 159.936]	<0.001	132.58 [73.299, 239.805]	<0.001
**75-79**	172.905 [102.285, 292.284]	<0.001	242.517 [134.141, 438.451]	<0.001
**80-84**	284.751 [168.501, 481.201]	<0.001	395.573 [218.839, 715.034]	<0.001
**85+**	539.705 [319.538, 911.572]	<0.001	725.971 [401.778, 1311.753]	<0.001
Pandemic				
**Pandemic**	1.001 [0.949, 1.055]	0.982	0.947 [0.887, 1.011]	0.101
Yearly Trend and Pandemic Interaction				
**Time * Pandemic**	1.005 [1.001, 1.009]	0.016	1.005 [1.001, 1.01]	0.025
NI: Not Included (the term was dropped during the backward selection)				
[a] P-values less than 0.001 are reported as '<0.001'.				

N00–N99 Diseases of the genitourinary system

	Female		Male	
	Estimate (95% CI)	P-Value[a]	Estimate (95% CI)	P-Value[a]
**Yearly Trend**				
**Time**	0.998 [0.997, 1]	0.013	0.999 [0.998, 1.001]	0.252
**Age Group**				
**30-34**	2.133 [1.113, 4.089]	0.023	1.722 [0.768, 3.864]	0.187
**35-39**	3.093 [1.678, 5.701]	<0.001	2.751 [1.306, 5.794]	0.008
**40-44**	3.775 [2.087, 6.828]	<0.001	7.105 [3.582, 14.093]	<0.001
**45-49**	7.737 [4.396, 13.619]	<0.001	10.049 [5.12, 19.722]	<0.001
**50-54**	14.669 [8.408, 25.593]	<0.001	23.686 [12.197, 45.998]	<0.001
**55-59**	24.34 [14.011, 42.284]	<0.001	36.742 [18.976, 71.141]	<0.001
**60-64**	46.876 [27.094, 81.101]	<0.001	77.629 [40.247, 149.734]	<0.001
**65-69**	90.341 [52.351, 155.899]	<0.001	149.854 [77.849, 288.459]	<0.001
**70-74**	180.86 [104.917, 311.772]	<0.001	274.67 [142.776, 528.405]	<0.001
**75-79**	403.348 [234.087, 694.997]	<0.001	548.442 [285.188, 1054.703]	<0.001
**80-84**	987.057 [573.001, 1700.314]	<0.001	1244.424 [647.243, 2392.598]	<0.001
**85+**	3615.292 [2099.168, 6226.437]	<0.001	3610.8 [1878.444, 6940.785]	<0.001
**Pandemic**				
**Pandemic**	1.024 [0.998, 1.051]	0.076	0.962 [0.382, 2.424]	0.934
**Yearly Trend and Pandemic Interaction**				
**Time * Pandemic**	1.004 [1.002, 1.006]	<0.001	1.004 [1.002, 1.006]	<0.001
**Age and Pandemic Interaction**				
**30-34 * Pandemic**	NI	NI	1.507 [0.495, 4.588]	0.47
**35-39 * Pandemic**	NI	NI	1.093 [0.382, 3.128]	0.868
**40-44 * Pandemic**	NI	NI	0.654 [0.245, 1.749]	0.398
**45-49 * Pandemic**	NI	NI	1.207 [0.466, 3.124]	0.699
**50-54 * Pandemic**	NI	NI	1.135 [0.444, 2.898]	0.791
**55-59 * Pandemic**	NI	NI	1.199 [0.472, 3.05]	0.703
**60-64 * Pandemic**	NI	NI	1.035 [0.409, 2.622]	0.941
**65-69 * Pandemic**	NI	NI	1.101 [0.436, 2.779]	0.839
**70-74 * Pandemic**	NI	NI	1.117 [0.443, 2.816]	0.815
**75-79 * Pandemic**	NI	NI	1.099 [0.436, 2.771]	0.841
**80-84 * Pandemic**	NI	NI	1.053 [0.418, 2.655]	0.912
**85+ * Pandemic**	NI	NI	1.049 [0.416, 2.642]	0.92
NI: Not Included (the term was dropped during the backward selection)				
[a] P-values less than 0.001 are reported as '<0.001'.				

O00–O99 Pregnancy, childbirth and the puerperium

	Female		Male	
	Estimate (95% CI)	P-Value[a]	Estimate (95% CI)	P-Value[a]
**Age Group**				
**30-34**	1.452 [0.84, 2.509]	0.182	NI	NI
**35-39**	1.484 [0.871, 2.529]	0.146	NI	NI
**40-44**	0.542 [0.283, 1.038]	0.065	NI	NI
**45-49**	0.06 [0.014, 0.257]	<0.001	NI	NI
**50-54**	0 [0, Inf]	0.993	NI	NI
**55-59**	0.037 [0.005, 0.278]	0.001	NI	NI
**60-64**	0 [0, Inf]	0.993	NI	NI
**65-69**	0 [0, Inf]	0.993	NI	NI
**70-74**	0.031 [0.004, 0.23]	0.001	NI	NI
**75-79**	0.038 [0.005, 0.279]	0.001	NI	NI
**80-84**	0 [0, Inf]	0.993	NI	NI
**85+**	0 [0, Inf]	0.993	NI	NI
NI: Not Included (the term was dropped during the backward selection)				
[a] P-values less than 0.001 are reported as '<0.001'.				

Q00–Q99 Congenital malformations, deformations and chromosomal abnormalities

	Female		Male	
	**Estimate (95% CI)**	**P-Value[a]**	**Estimate (95% CI)**	**P-Value[a]**
**Yearly Trend**				
**Time**	1.011 [0.967, 1.057]	0.634	NI	NI
**Age Group**				
**30-34**	1.484 [0.631, 3.491]	0.365	0.989 [0.694, 1.408]	0.951
**35-39**	0.959 [0.399, 2.308]	0.926	1.174 [0.842, 1.636]	0.344
**40-44**	1.183 [0.513, 2.726]	0.694	0.971 [0.694, 1.358]	0.861
**45-49**	0.986 [0.431, 2.253]	0.973	1.229 [0.898, 1.681]	0.198
**50-54**	1.152 [0.511, 2.597]	0.733	1.578 [1.161, 2.146]	0.004
**55-59**	4.387 [2.078, 9.263]	<0.001	2.316 [1.724, 3.111]	<0.001
**60-64**	2.891 [1.357, 6.162]	0.006	2.591 [1.934, 3.471]	<0.001
**65-69**	3.734 [1.794, 7.775]	<0.001	3.107 [2.342, 4.122]	<0.001
**70-74**	3.241 [1.548, 6.784]	0.002	2.526 [1.891, 3.373]	<0.001
**75-79**	3.626 [1.748, 7.522]	0.001	3.729 [2.802, 4.964]	<0.001
**80-84**	5.623 [2.732, 11.572]	<0.001	5.6 [4.216, 7.438]	<0.001
**85+**	12.459 [6.246, 24.855]	<0.001	10.913 [8.314, 14.326]	<0.001
**Pandemic**				
**Pandemic**	0.664 [0.196, 2.256]	0.512	NI	NI
**Age and Time Interaction**				
**30-34 * Time**	0.999 [0.944, 1.056]	0.963	NI	NI
**35-39 * Time**	0.984 [0.93, 1.041]	0.58	NI	NI
**40-44 * Time**	0.996 [0.943, 1.052]	0.884	NI	NI
**45-49 * Time**	0.965 [0.915, 1.018]	0.187	NI	NI
**50-54 * Time**	0.975 [0.925, 1.027]	0.338	NI	NI
**55-59 * Time**	1.035 [0.985, 1.089]	0.175	NI	NI
**60-64 * Time**	1.002 [0.953, 1.053]	0.943	NI	NI
**65-69 * Time**	1.008 [0.96, 1.057]	0.758	NI	NI
**70-74 * Time**	0.999 [0.952, 1.049]	0.976	NI	NI
**75-79 * Time**	0.982 [0.936, 1.03]	0.446	NI	NI
**80-84 * Time**	0.989 [0.943, 1.037]	0.642	NI	NI
**85+ * Time**	0.986 [0.942, 1.031]	0.528	NI	NI
**Age and Pandemic Interaction**				
**30-34 * Pandemic**	1.031 [0.218, 4.866]	0.969	NI	NI
**35-39 * Pandemic**	1.881 [0.397, 8.911]	0.426	NI	NI
**40-44 * Pandemic**	1.42 [0.317, 6.359]	0.647	NI	NI
**45-49 * Pandemic**	2.054 [0.475, 8.887]	0.335	NI	NI
**50-54 * Pandemic**	2.541 [0.604, 10.69]	0.203	NI	NI
**55-59 * Pandemic**	0.42 [0.105, 1.674]	0.219	NI	NI
**60-64 * Pandemic**	1.189 [0.302, 4.674]	0.804	NI	NI
**65-69 * Pandemic**	0.937 [0.247, 3.557]	0.924	NI	NI
**70-74 * Pandemic**	0.976 [0.255, 3.731]	0.971	NI	NI

**75-79 * Pandemic**	1.935 [0.521, 7.184]	0.324	NI	NI
**80-84 * Pandemic**	1.591 [0.432, 5.853]	0.485	NI	NI
**85+ * Pandemic**	1.596 [0.457, 5.575]	0.464	NI	NI
NI: Not Included (the term was dropped during the backward selection)				
[a] P-values less than 0.001 are reported as '<0.001'.				

S00–T98 V01–Y98 Injury, poisoning and certain other consequences of external causes

	Female		Male	
	Estimate (95% CI)	P-Value[a]	Estimate (95% CI)	P-Value[a]
**Yearly Trend**				
**Time**	1.003 [1, 1.007]	0.058	0.997 [0.995, 1]	0.019
**Age Group**				
**30-34**	0.868 [0.812, 0.928]	<0.001	1.069 [1.025, 1.115]	0.002
**35-39**	0.879 [0.824, 0.938]	<0.001	1.071 [1.028, 1.116]	0.001
**40-44**	0.924 [0.869, 0.983]	0.013	1.13 [1.086, 1.175]	<0.001
**45-49**	1.103 [1.041, 1.169]	0.001	1.257 [1.211, 1.306]	<0.001
**50-54**	1.287 [1.215, 1.364]	<0.001	1.498 [1.443, 1.555]	<0.001
**55-59**	1.343 [1.267, 1.424]	<0.001	1.728 [1.664, 1.794]	<0.001
**60-64**	1.456 [1.374, 1.542]	<0.001	1.867 [1.799, 1.938]	<0.001
**65-69**	1.831 [1.734, 1.934]	<0.001	2.219 [2.142, 2.3]	<0.001
**70-74**	2.775 [2.635, 2.922]	<0.001	2.947 [2.847, 3.05]	<0.001
**75-79**	4.633 [4.406, 4.872]	<0.001	4.512 [4.361, 4.668]	<0.001
**80-84**	7.767 [7.394, 8.16]	<0.001	7.363 [7.12, 7.614]	<0.001
**85+**	18.573 [17.713, 19.474]	<0.001	15.591 [15.096, 16.102]	<0.001
**Pandemic**				
**Pandemic**	1.048 [1.022, 1.074]	<0.001	1.042 [1.022, 1.063]	<0.001
**Yearly Trend and Pandemic Interaction**				
**Time * Pandemic**	1.008 [1.006, 1.009]	<0.001	1.005 [1.003, 1.006]	<0.001
**Age and Time Interaction**				
**30-34 * Time**	0.995 [0.991, 1]	0.064	0.997 [0.994, 1]	0.038
**35-39 * Time**	0.995 [0.99, 1]	0.033	0.998 [0.995, 1.001]	0.172
**40-44 * Time**	0.994 [0.99, 0.999]	0.009	0.998 [0.996, 1.001]	0.274
**45-49 * Time**	0.992 [0.988, 0.996]	<0.001	0.998 [0.995, 1]	0.089
**50-54 * Time**	0.991 [0.987, 0.995]	<0.001	0.995 [0.992, 0.997]	<0.001
**55-59 * Time**	0.993 [0.988, 0.997]	0.001	0.996 [0.993, 0.998]	0.001
**60-64 * Time**	0.991 [0.987, 0.995]	<0.001	0.995 [0.992, 0.998]	<0.001
**65-69 * Time**	0.987 [0.983, 0.99]	<0.001	0.994 [0.992, 0.997]	<0.001
**70-74 * Time**	0.987 [0.984, 0.991]	<0.001	0.995 [0.993, 0.998]	<0.001
**75-79 * Time**	0.987 [0.983, 0.99]	<0.001	0.996 [0.994, 0.999]	0.002
**80-84 * Time**	0.986 [0.982, 0.989]	<0.001	0.995 [0.993, 0.997]	<0.001
**85+ * Time**	0.989 [0.986, 0.992]	<0.001	0.997 [0.995, 1]	0.029
NI: Not Included (the term was dropped during the backward selection)				
[a] P-values less than 0.001 are reported as '<0.001'.				
